# Traumatic Spine Injury in Southern Ethiopia: Falls, Delayed Presentation, and High Early Mortality at a Tertiary Referral Center

**DOI:** 10.3390/jcm15093276

**Published:** 2026-04-25

**Authors:** Mengistu G. Mengesha, Sultan Baz, Hermella Damenu, Hana-Joy Hanks, Ryan Beyer, Alexander Nazareth, Sohaib Hashmi, Hao-Hua Wu

**Affiliations:** 1 Hawassa University Comprehensive Specialized Hospital, Hawassa P.O. Box 1560, Ethiopia; mengistugy@gmail.com (M.G.M.); spineresearch024@gmail.com (H.D.); 2 Global Spine Research Initiative, Department of Orthopaedic Surgery, University of California, Irvine, CA 92697, USA; hanksh@hs.uci.edu (H.-J.H.); beyerrs@hs.uci.edu (R.B.); nazaret1@hs.uci.edu (A.N.); szhashmi@hs.uci.edu (S.H.); haohuaw1@hs.uci.edu (H.-H.W.)

**Keywords:** traumatic spine injury, spinal cord injury, Ethiopia, low- and middle-income countries, epidemiology, mortality, referral delay, trauma registry

## Abstract

**Background/Objectives**: Traumatic spine injury is a major cause of morbidity and mortality in low- and middle-income countries, yet detailed epidemiologic data from sub-Saharan Africa remain limited. We used a fracture registry to characterize injury patterns, care pathways, and short-term outcomes among patients presenting with traumatic spine injury at a tertiary referral center in Ethiopia. **Methods**: We performed a retrospective analysis of a prospectively maintained fracture registry at a tertiary referral hospital in Ethiopia from June 2023 to July 2025. Patients with traumatic spine injury were included. Variables included demographics, injury mechanism and context, injury region, AO morphology, neurologic status (ASIA), referral status, mode of transportation, time to presentation, treatment, and 30-day outcomes. Descriptive statistics were used to summarize the cohort. Bivariate associations were assessed using chi-square or Fisher’s exact tests, and crude odds ratios were calculated for prespecified 2 × 2 comparisons. **Results**: A total of 252 patients were included (mean age: 33.1 ± 13.6 years; 81.3% male). Falls (45.2%) and road traffic accidents (26.2%) were the most common mechanisms, and injuries most often occurred on farms (40.1%) and roads/streets (33.7%). The thoracolumbar (31.3%) and cervical (30.6%) regions were most frequently affected. Complete spinal cord injury (ASIA A) occurred in 36.5% of patients. Most patients were referred (88.5%), 62.7% presented >24 h after injury, and 65.5% were managed non-operatively. Referral status was strongly associated with delayed presentation (OR: 10.49, 95% CI: 3.84–28.64). Thirty-day mortality was 22.2%. Complete SCI (OR: 6.17, 95% CI: 3.23–11.90) and cervical/thoracic injuries (OR: 6.54, 95% CI: 3.12–13.70) were associated with higher mortality. **Conclusions**: Traumatic spine injury in this Ethiopian cohort disproportionately affected young adults and was marked by severe neurologic injury, delayed presentation, and high early mortality.

## 1. Introduction

Traumatic spine injury is a devastating global health issue characterized by profound health loss from premature mortality and chronic disability [1]. The Global Burden of Disease Study 2019 reports that over 20.6 million individuals are currently living with spinal cord injuries, with absolute case numbers increasing substantially over the past three decades [1]. This burden is disproportionately concentrated in low- and middle-income countries where the incidence is estimated at 13.69 per 100,000 people, significantly higher than the 8.72 per 100,000 observed in high-income countries [2,3]. Systematic reviews of low- and middle-income countries reveal a pooled traumatic spinal cord injury (SCI) incidence of 22.55 cases per million annually, with males consistently comprising more than 80% of victims [4,5].

In Sub-Saharan Africa (SSA), the epidemiology of spine trauma is defined by severe energy mechanisms and significant resource scarcity [6,7]. Systematic reviews across the continent report that acute mortality rates from traumatic spine injuries range from 18% to 25%, a stark contrast to the near-zero mortality often seen in high-income settings [2,7]. Etiological profiles in SSA are diverse; while road traffic accidents (RTAs) are a primary driver, high-energy falls and interpersonal violence are increasingly prevalent causes of catastrophic cord disruption [7,8,9]. In Ethiopia, traumatic spine injury disproportionately affects young, working-age adults and represents an important cause of disability, yet detailed longitudinal data from regions outside the capital remain limited [10,11,12].

The management of spine trauma in regions like Ethiopia is complicated by profound pre-hospital delays and fragmented referral systems [13,14,15]. Victims often lack access to specialized immobilization and rely on non-ambulance transportation, such as public transit, which can exacerbate neurological deficits during transport [15,16]. Furthermore, the financial risk of neurosurgical care is immense; out-of-pocket expenses for surgery and implants are often catastrophic for families in SSA, where social determinants of health like insurance status and geographic accessibility strongly dictate outcomes [17,18,19]. In Ethiopia, these challenges occur within a health system marked by limited prehospital resources, fragmented interfacility referral pathways, and substantial financial barriers to definitive care [13,14,15,16,17,18,19]. These systemic barriers lead to a “referral-delay” paradox, where patients are referred through multiple facilities but experience prolonged wait times that miss the optimal surgical window for decompression [20,21]. Together, these factors highlight the need for region-specific epidemiologic data to inform prevention strategies, referral system improvement, and trauma care planning. This study provides a comprehensive analysis of a spine trauma cohort, with or without SCI (n = 252), at a tertiary referral center in Hawassa, Southern Ethiopia, to characterize injury patterns, care pathways, and short-term outcomes in this setting and to address an important regional data gap.

## 2. Materials and Methods

We conducted a retrospective analysis of a prospectively maintained fracture registry at Hawassa University Comprehensive Specialized Hospital. This facility serves as a primary regional hub for specialized neurosurgical and orthopedic care in East Africa [10,22]. The study period spanned from June 2023 through July 2025. The Institutional Review Board of Hawassa University approved this research.

The cohort included all patients presenting with traumatic spine injuries confirmed through clinical and radiographic examination. We excluded patients with non-traumatic spine pathologies or those with incomplete records lacking essential injury data or 30-day outcome data [23,24].

Data were extracted to characterize demographics (age, sex, and residence), socioeconomic factors (occupation and insurance status), and injury-specific characteristics. Fracture morphology was classified using the AO Spine Classification System [3,25]. Neurological status at admission was graded using the American Spinal Injury Association Impairment Scale [4,5]. System-level variables included referral status, mode of transportation, and the time interval from injury to presentation [1,7].

Statistical analyses were conducted using IBM SPSS Statistics for Windows, version 31.0 (IBM Corp., Armonk, NY, USA). Continuous variables were summarized as mean ± standard deviation (SD) or median with interquartile range (IQR), as appropriate based on distribution. Categorical variables were summarized using frequencies and percentages. Bivariate associations were assessed using chi-square tests. To quantify the magnitude of these associations, effect size measures were employed [26]. For 2 × 2 tables, the Phi coefficient was utilized [27]. For larger tables, Cramér’s V was applied [28]. Both coefficients range from 0 to 1, where 0 indicates no association and 1 indicates a perfect association [29]. The strength of association was interpreted as negligible (0.00–0.10), weak (0.10–0.30), moderate (0.30–0.50), or strong (>0.50) [26,30]. Crude odds ratios with 95% confidence intervals were calculated to identify factors associated with mortality and delay [31].

Multivariable binary logistic regression analyses were performed to identify factors independently associated with 30-day mortality and delayed presentation (>24 h). Covariates were selected a priori based on clinical relevance and included age, sex, neurologic severity, injury level, AO classification, referral status, and insurance status. Neurologic severity was dichotomized as ASIA A versus non-ASIA A, and injury level was grouped as cervical/thoracic versus thoracolumbar/lumbar. Adjusted odds ratios (aORs) with 95% confidence intervals were reported. Model fit was assessed using the Hosmer–Lemeshow goodness-of-fit test and Nagelkerke R^2^.

Because this was a retrospective registry-based study, no a priori sample size calculation was performed. All eligible patients presenting during the predefined study period were included in the analysis.

## 3. Results

### 3.1. Cohort Characteristics and Clinical Outcomes

#### 3.1.1. Sociodemographic Profile

A total of 252 patients were included. The cohort was predominantly young and male, with a mean age of 33.1 ± 13.6 years and 81.3% identified as male [5,8,24]. Urban residents comprised 59.1% of the cohort, while 40.9% resided in rural areas. Financial vulnerability was significant: 65.1% of patients were uninsured, and 54.0% relied on informal financial support [17,32]. Baseline demographic, socioeconomic, injury, and care-pathway characteristics are summarized in Table 1.

#### 3.1.2. Injury Etiology and Severity

**Figure 1:**
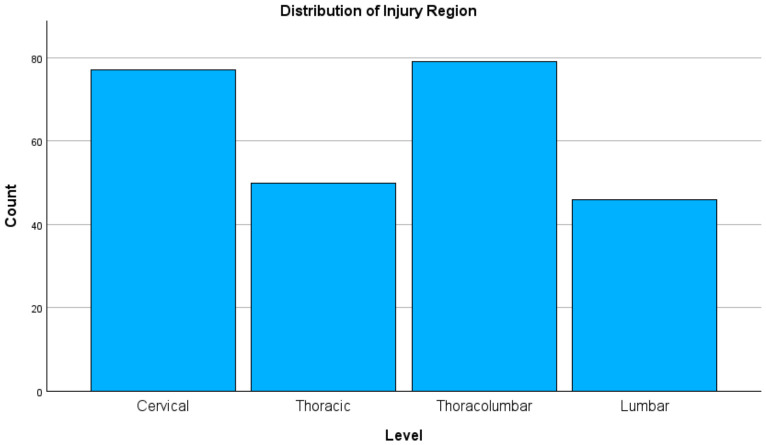
Distribution of injury mechanisms among patients with traumatic spine injury.

**Figure 2:**
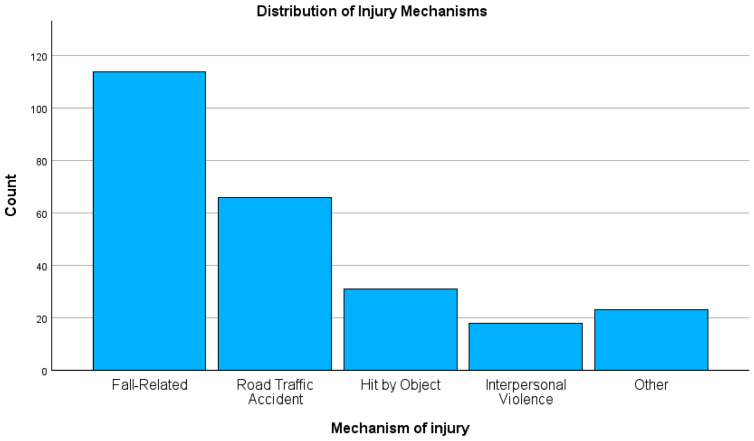
Distribution of injury regions among patients with traumatic spine injury.

High-energy mechanisms accounted for a significant portion of all injuries. Falls were the most common mechanism of injury. RTAs were the second most common cause (26.2%) [8,25,33] (Figure 1). The thoracolumbar region (T12–L1) was most frequently affected region (31.3%), followed by the cervical spine (30.6%). According to the AO classification, Type C (translational) injuries were highly prevalent (39.3%) [34] (Figure 2).

#### 3.1.3. Neurological Status and Delays

Neurological impairment was severe: 36.5% of patients had complete SCI, and 35.7% had incomplete deficits [4,5]. AO morphology was significantly associated with neurological severity (*p* < 0.001), demonstrating a moderate effect size (Cramér’s V = 0.358). Systemic barriers were profound: median time from injury to presentation was 48.0 h (IQR: 16.25–120.0 h), 88.5% of patients were referred from another facility, and 62.7% experienced a presentation delay of >24 h. Referral status was significantly associated with delayed presentation (*p* < 0.001; Phi = 0.339). Referred patients had over 10 times higher odds of arriving > 24 h (OR: 10.49; 95% CI: 3.84–28.64) [21,35]. Bivariate associations among injury characteristics, neurologic severity, delayed presentation, and 30-day mortality are summarized in Table 2.

#### 3.1.4. 30-Day Mortality

**Figure 3:**
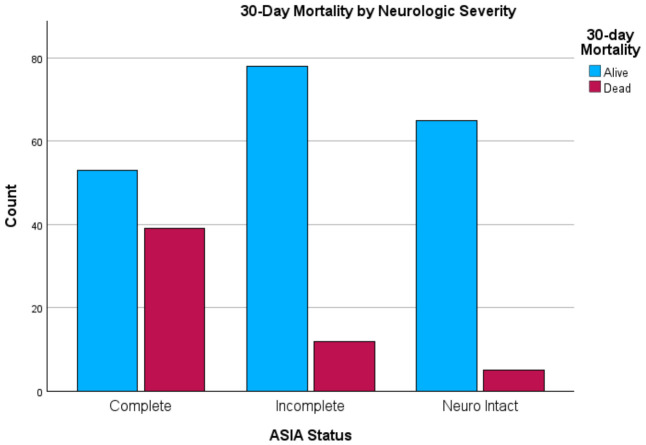
Thirty-day outcome by neurologic severity at presentation.

The overall 30-day mortality rate was 22.2%. Mortality was strongly associated with neurological severity (*p* < 0.001; Cramér’s V = 0.373) (Figure 3). Patients with complete SCI had 6.17 times higher odds of death (OR: 6.17; 95% CI: 3.23–11.90) [10,31]. Injury level (cervical/thoracic) was also a significant predictor of mortality (OR: 6.54; 95% CI: 3.12–13.70; Cramér’s V = 0.340). Mortality was significantly lower in surgically managed patients (6.9%) compared to non-operative cases (30.3%; *p* < 0.001; Phi = 0.268) [10,22]. Crude odds ratios for delayed presentation and 30-day mortality are summarized in Table 3.

Multivariable logistic regression was performed to identify factors independently associated with 30-day mortality. The overall model was significant (omnibus χ^2^ = 65.83, df = 8, *p* < 0.001), demonstrated acceptable calibration (Hosmer–Lemeshow *p* = 0.874), and explained a moderate proportion of outcome variance (Nagelkerke R^2^ = 0.352). After adjustment for age, sex, AO classification, referral status, and insurance status, complete SCI (ASIA A) remained independently associated with higher odds of 30-day mortality (aOR: 6.49, 95% CI: 2.95–14.27; *p* < 0.001), as did cervical/thoracic injury level compared with thoracolumbar/lumbar injury level (aOR: 5.84, 95% CI: 2.61–13.04; *p* < 0.001). Age, sex, AO classification, referral status, and insurance status were not independently associated with 30-day mortality in the adjusted model.

A second multivariable logistic regression model was performed to identify factors independently associated with delayed presentation (>24 h). The model was significant overall (omnibus χ^2^ = 37.89, df = 8, *p* < 0.001), demonstrated acceptable calibration (Hosmer–Lemeshow *p* = 0.830), and explained a modest proportion of the variance (Nagelkerke R^2^ = 0.190). After adjustment, referral status remained independently associated with delayed presentation (aOR: 9.77, 95% CI: 3.50–27.22; *p* < 0.001). Female sex was also independently associated with delayed presentation (aOR: 2.53, 95% CI: 1.14–5.62; *p* = 0.023). Age, neurologic severity, injury level, AO classification, and insurance status were not independently associated with delayed presentation in the adjusted model. The multivariable logistic regression models for 30-day mortality and delayed presentation are summarized in Table 4.

To improve readability, selected key descriptive findings were also presented graphically, including injury mechanism distribution, injury region distribution, and 30-day mortality by neurologic severity.

## 4. Discussion

The findings from this Ethiopian cohort highlight a public health crisis affecting the nation’s most productive demographic. Our cohort demonstrated a mean age of 33.1 years and an 81.3% male predominance, mirroring trends in other LMICs where young men are disproportionately affected due to high-risk occupational roles [4,5,36]. Multisite surveillance in Kenya has similarly identified traumatic injuries as a leading cause of death among young men [24]. The socioeconomic fallout is profound; the loss of a primary breadwinner to permanent disability often triggers multi-generational poverty [2,37,38]. Covell et al. emphasize that Social Determinants of Health (SDoH), including income and education, are critical drivers of post-injury mortality in these settings [17,19].

A critical observation is the predominance of falls (45.2%) over RTAs (26.2%). While broader African meta-analyses often cite RTAs as the primary etiology, our results align with emerging data from the Ethiopian highlands where falls from construction sites and trees are the leading mechanism [8,25,39]. This pattern likely reflects the occupational and economic structure of Southern Ethiopia, where agricultural labor and other forms of manual work are common and may increase exposure to fall-related trauma. In our cohort, nearly half of patients were engaged in agriculture/manual labor, and a substantial proportion of injuries occurred on farms, supporting the interpretation that region-specific occupational exposures contribute meaningfully to the observed etiologic profile. In addition, informal construction work and limited workplace safety protections may further increase the risk of high-energy falls in this setting. In this regional context, agricultural activities and unregulated construction are significant drivers of high-energy trauma [11,33]. Similar patterns have been observed in rural Uganda and Kenya, where falls have surpassed RTAs in specific referral cohorts [40,41]. Taken together, these findings suggest that prevention priorities in Southern Ethiopia may need to extend beyond road safety alone and include occupational injury prevention strategies tailored to agricultural and informal labor settings. This highlights an urgent need for occupational safety regulations targeted at these high-risk activities.

Our study identified a critical 62.7% rate of delayed presentation. The “referral-delay” paradox, where referred patients were 10.49 times more likely to arrive late, is a hallmark of fragmented trauma networks in SSA [20,21,24]. Furthermore, 49.2% of patients used non-ambulance transportation. This reliance on untrained bystanders is a known risk factor for neurological deterioration, as research in Malawi highlighted that a lack of formal EMS infrastructure significantly impedes acute care [15,23,35]. Addressing these systemic delays through regionalized trauma protocols is essential for improving African spine trauma outcomes [25]. Although delayed presentation was not significantly associated with 30-day mortality in our cohort, this finding should be interpreted cautiously. Survivorship bias may have influenced this result, as patients with the most severe injuries may have died before reaching the hospital, whereas those presenting after prolonged delays were, by definition, stable enough to survive the initial post-injury period and transport.

The overall mortality rate of 22.2% aligns with the upper range reported across SSA [2,7]. Bivariate analysis showed that cervical/thoracic injury level (OR: 6.54) and complete SCI (OR: 6.17) were significantly associated with 30-day mortality (both *p* < 0.001). These findings are consistent with studies identifying high neurological grade as a primary driver of fatal pulmonary complications and sepsis in resource-constrained settings [22,31,42]. Although operative management was associated with lower crude 30-day mortality, this result should be interpreted cautiously as the non-operative group may have disproportionately included patients who died early or were too unstable to undergo surgery. Even for survivors, the long-term prognosis in LMICs is often poor due to a lack of community-based rehabilitation and SDoH barriers [17,32].

This study is a single-center retrospective analysis, and the findings may not be fully generalizable to the broader Ethiopian population or to patients who never reached tertiary care [35,43]. Because the cohort was derived from a tertiary referral registry, selection bias is possible, with potential overrepresentation of patients with more severe injuries who survived long enough to reach specialized care. Conversely, patients who died before transfer or were never referred would not have been captured, which may have influenced estimates of injury severity, delays, and mortality. Information bias is also possible because registry-based retrospective analyses depend on the completeness and accuracy of recorded clinical, referral, transport, and treatment data, some of which were not uniformly documented across patients. The data are further restricted to 30-day outcomes, precluding assessment of long-term neurologic recovery, functional status, and post-discharge mortality [17,32]. In addition, no a priori sample size calculation was performed, and some subgroup comparisons may have been underpowered, particularly for less frequent exposure categories. Future research should include prospective multicenter studies with more granular referral and transport data, clearer documentation of treatment-selection factors, and longitudinal follow-up to better define long-term outcomes after traumatic spine injury in Ethiopia and similar settings.

Although referral status was strongly associated with delayed presentation, the registry did not consistently capture the number of prior facilities visited, transfer intervals, or the specific logistical reasons for transfer, which limits deeper causal interpretation of the observed referral-delay paradox. Likewise, although operative management was associated with lower crude 30-day mortality, treatment allocation in this setting was likely influenced by factors such as physiologic stability, neurologic severity, surgical candidacy, implant availability, transfer timing, and financial barriers. Accordingly, this association should not be interpreted as evidence of a direct protective effect of surgery.

The registry also lacked sufficiently granular referral data, including transfer sequence, referral timing, and transport-specific decision points, which limited our ability to identify the precise mechanisms underlying delayed presentation. In addition, the specific clinical and systems-level reasons for non-operative management were not consistently recorded, precluding a more definitive analysis of treatment selection and its relationship to outcomes.

In addition, no a priori sample size calculation was performed because this was a retrospective analysis of an existing registry. Although the overall cohort size was adequate for the primary descriptive aims and multivariable analyses performed, some subgroup comparisons may have been underpowered, particularly for less frequent exposure categories, and non-significant findings should therefore be interpreted cautiously.

## 5. Conclusions

Traumatic spine injury in this Ethiopian cohort disproportionately affected young adults, characterized by high-energy mechanisms, severe fracture morphology, and frequent complete neurological impairment [7,25]. The predominance of falls from height (45.2%) highlights a distinct occupational hazard in the Southern Ethiopian context [11,33]. Systemic barriers were profound, evidenced by a 62.7% rate of delayed presentation and a referral-delay paradox [20,21]. The high 30-day mortality rate (22.2%) was independently associated with complete SCI and cervical/thoracic injury level in adjusted analyses [7,31]. These findings underscore the need for practical, region-specific interventions rather than broad policy goals alone. Potential priorities include standardized referral and transfer pathways, improved access to prehospital immobilization and ambulance transport, and occupational injury-prevention strategies targeting agricultural and informal labor settings. Such measures may help reduce delays in care, limit secondary neurologic injury, and improve early outcomes after traumatic spine injury in Southern Ethiopia and similar resource-constrained settings [1,2].

## Figures and Tables

**Table 1:** Baseline Demographic, Injury, and Care-Pathway Characteristics of Patients With Traumatic Spine Injury At A Tertiary Referral Center in Ethiopia.

Characteristic	n	Value
Continuous variables		
Age, years	252	33.1 ± 13.6
Time to presentation, h	252	48.0 (16.25–120.0)
Length of stay, days	114	14.5 ± 11.7
Demographics and socioeconomic factors		
Sex—Male	205	81.3%
Sex—Female	47	18.7%
Residence—Rural	103	40.9%
Residence—Urban	149	59.1%
Occupation—Agriculture/manual labor	125	49.6%
Occupation—Student	42	16.7%
Occupation—Self-employed	53	21.0%
Occupation—Non-labor	32	12.7%
Education—No formal education	86	34.1%
Education—Primary	105	41.7%
Education—Secondary or above	61	24.2%
Insurance—Uninsured	164	65.1%
Insurance—Insured	88	34.9%
Injury and care-pathway characteristics		
Mechanism—Fall-related (height or ground-level)	114	45.2%
Mechanism—Road traffic accident	66	26.2%
Mechanism—Hit by object	31	12.3%
Mechanism—Interpersonal violence	18	7.1%
Mechanism—Other	23	9.1%
Delayed presentation (>24 h)—Early	94	37.3%
Delayed presentation (>24 h)—Delayed	158	62.7%
30-day outcome—Dead	56	22.2%
30-day outcome—Alive	196	77.8%
Definitive management—Non-operative	165	65.5%
Definitive management—Operative	87	34.5%
Pelvic injury—No	237	94.0%
Pelvic injury—Yes	15	6.0%
Mode of transportation—Ambulance	118	46.8%
Mode of transportation—Non-ambulance	124	49.2%
Mode of transportation—Carried/walked	10	4.0%
ASIA classification—Complete SCI (A)	92	36.5%
ASIA classification—Incomplete SCI (B–D)	90	35.7%
ASIA classification—Neurologically intact (E)	70	27.8%
Injury region—Cervical	77	30.6%
Injury region—Thoracic	50	19.8%
Injury region—Thoracolumbar (T12–L1)	79	31.3%
Injury region—Lumbar	46	18.3%
AO classification—Type A	100	39.7%
AO classification—Type B	53	21.0%
AO classification—Type C	99	39.3%
Nature of injury—Closed	240	95.2%
Nature of injury—Open	12	4.8%
Associated injury—No	144	57.1%
Associated injury—Yes	108	42.9%
Place of injury—Farm	101	40.1%
Place of injury—Home	37	14.7%
Place of injury—Road/street	85	33.7%
Place of injury—Construction area	10	4.0%
Place of injury—Community/other	19	7.5%
TBS visit—No	219	86.9%
TBS visit—Yes	33	13.1%
Referral status—No	29	11.5%
Referral status—Yes	223	88.5%
Admission status—No	154	61.1%
Admission status—Yes	98	38.9%
Comorbidities—No	244	96.8%
Comorbidities—Yes	8	3.2%

**Table 2:** Bivariate Associations Between Injury and System Factors and Neurologic Severity, Delayed Presentation, and 30-Day Mortality among Patients with Traumatic Spine Injury (N = 252). (**A**) Variables vs. neurologic severity (ASIA 3-category); (**B**) Mechanism of injury (5-level) vs. injury region (C/T/TL/L); (**C**) Variables vs. delayed presentation (>24 h); (**D**) Variables vs. 30-day mortality.

(**A**)							
**Variable**	**Complete SCI**	**Incomplete SCI**		**Intact**	**χ^2^**	***p*** **-Value**	**Cramér’s V**
AO type					64.471	<0.001	0.358
Type A	23 (23.0%)	32 (32.0%)		45 (45.0%)			
Type B	7 (13.2%)	27 (50.9%)		19 (35.8%)			
Type C	62 (62.6%)	31 (31.3%)		6 (6.1%)			
Injury region					24.399	<0.001	0.220
Cervical	25 (32.5%)	29 (37.7%)		23 (29.9%)			
Thoracic	29 (58.0%)	13 (26.0%)		8 (16.0%)			
Thoracolumbar	32 (40.5%)	29 (36.7%)		18 (22.8%)			
Lumbar	6 (13.0%)	19 (41.3%)		21 (45.7%)			
(**B**)							
**Mechanism**	**Cervical**	**Thoracic**	**Thoracolumbar**	**Lumbar**	**χ^2^**	***p*** **-Value**	**Cramér’s V**
Fall-related	14 (12.3%)	34 (29.8%)	47 (41.2%)	19 (16.7%)	68.478	0.001	0.301
Road traffic accident	29 (43.9%)	7 (10.6%)	15 (22.7%)	15 (22.7%)			
Hit by object	9 (29.0%)	6 (19.4%)	12 (38.7%)	4 (12.9%)			
Interpersonal violence	6 (33.3%)	3 (16.7%)	2 (11.1%)	7 (38.9%)			
Other	19 (82.6%)	0 (0.0%)	3 (13.0%)	1 (4.3%)			
(**C**)							
**Variable**	**Early**		**Delayed**		**χ^2^**	***p*** **-Value**	**Phi**
Transport mode					4.850	0.088	0.139
Ambulance	50 (42.4%)		68 (57.6%)				
Non-ambulance	43 (34.7%)		81 (65.3%)				
Carried/walked	1 (10.0%)		9 (90.0%)				
Referral status					28.954	<0.001	0.339
Not referred	24 (82.8%)		5 (17.2%)				
Referred	70 (31.4%)		153 (68.6%)				
(**D**)							
**Variable**	**Dead**		**Alive**		**χ^2^**	***p*** **-Value**	**Cramér’s V/Phi**
ASIA grade					34.976	<0.001	0.373
Complete SCI	39 (42.4%)		53 (57.6%)				
Incomplete SCI	12 (13.3%)		78 (86.7%)				
Intact	5 (7.1%)		65 (92.9%)				
Injury region					29.121	<0.001	0.340
Cervical	28 (36.4%)		49 (63.6%)				
Thoracic	18 (36.0%)		32 (64.0%)				
Thoracolumbar	7 (8.9%)		72 (91.1%)				
Lumbar	3 (6.5%)		43 (93.5%)				
AO type					14.276	<0.001	0.238
Type A	19 (19.0%)		81 (81.0%)				
Type B	4 (7.5%)		49 (92.5%)				
Type C	33 (33.3%)		66 (66.7%)				
Time to presentation					0.438	0.508	0.042
Early	23 (24.5%)		71 (75.5%)				
Delayed	33 (20.9%)		125 (79.1%)				
Definitive management					18.056	<0.001	0.268
Non-operative	50 (30.3%)		115 (69.7%)				
Operative	6 (6.9%)		81 (93.1%)				

**Table 3:** Crude Odds Ratios For Delayed Presentation (>24 h) and 30-Day Mortality Among Patients with Traumatic Spine Injury (N = 252).

Exposure (Reference)	Outcome	Crude OR	95% CI
Referred vs. not referred	Delayed presentation (>24 h)	10.49	3.84–28.64
ASIA A vs. ASIA B–E	30-day mortality	6.17	3.23–11.90
Cervical/thoracic vs. thoracolumbar/lumbar	30-day mortality	6.54	3.12–13.70

**Table 4:** Multivariable Logistic Regression Analyses for 30-Day Mortality and Delayed Presentation.

Predictor	30-Day Mortality aOR (95% CI)	*p*-Value	Delayed Presentation aOR (95% CI)	*p*-Value
Age	1.01 (0.99–1.04)	0.394	0.99 (0.97–1.01)	0.419
Female sex (vs. male)	1.45 (0.58–3.62)	0.425	2.53 (1.14–5.62)	0.023
ASIA A (vs. non-ASIA A)	6.49 (2.95–14.27)	<0.001	1.01 (0.52–1.96)	0.970
Cervical/thoracic injury (vs. thoracolumbar/lumbar)	5.84 (2.61–13.04)	<0.001	1.06 (0.59–1.88)	0.854
AO Type B (vs. Type A)	0.50 (0.14–1.78)	0.282	0.62 (0.29–1.32)	0.212
AO Type C (vs. Type A)	1.00 (0.46–2.20)	0.993	0.93 (0.47–1.85)	0.830
Referred (vs. not referred)	0.86 (0.29–2.50)	0.777	9.77 (3.50–27.22)	<0.001
Insured (vs. uninsured)	0.52 (0.23–1.17)	0.116	1.58 (0.86–2.91)	0.137

Abbreviations: aOR, adjusted odds ratio; CI, confidence interval; ASIA, American Spinal Injury Association. Reference categories: male sex, non-ASIA A, thoracolumbar/lumbar injury, AO Type A, not referred, and uninsured. Model fit: 30-day mortality model, Nagelkerke R^2^ = 0.352, Hosmer–Lemeshow *p* = 0.874; delayed-presentation model, Nagelkerke R^2^ = 0.190, Hosmer–Lemeshow *p* = 0.830.

## Data Availability

The data presented in this study are available from the corresponding author on reasonable request. The data are not publicly available due to privacy and institutional restrictions.

## Abbreviations

The following abbreviations are used in this manuscript:

AO	Arbeitsgemeinschaft für Osteosynthesefragen
ASIA	American Spinal Injury Association
CI	confidence interval
LMICs	low- and middle-income countries
OR	odds ratio
RTA	road traffic accident
SCI	spinal cord injury
SD	standard deviation
